# Incidence of infective endocarditis caused by viridans group streptococci in Sweden – effect of cessation of antibiotic prophylaxis in dentistry for risk individuals

**DOI:** 10.1080/20002297.2020.1768342

**Published:** 2020-05-23

**Authors:** Niko Vähäsarja, Bodil Lund, Anders Ternhag, Bengt Götrick, Lars Olaison, Margareta Hultin, Carina Krüger Weiner, Aron Naimi-Akbar

**Affiliations:** a Department of Dental Medicine, Karolinska Institutet, Huddinge, Sweden; b Folktandvården Stockholms Län AB, Folktandvården Eastmaninstitutet; c Department of Clinical Dentistry, University of Bergen, Bergen, Norway; d Department of Oral and Maxillofacial Surgery, Haukelands University Hospital, Bergen, Norway; e Department of Medicine Solna, Karolinska Institutet, Unit for Infectious Diseases, Karolinska University Hospital, Stockholm, Sweden; f Department of Oral Diagnostics Faculty of Odontology, Malmö University, Malmö, Sweden; g Department of Infectious Diseases, Institute of Biomedicine, Sahlgrenska University Hospital, Sweden; h Health Technology Assessment-Odontology (HTA-O), Malmö University, Malmö, Sweden

**Keywords:** Prophylactic antibiotics, dentistry, infective endocarditis, viridans group streptococci

## Abstract

**Introduction:**

In October 2012, the Swedish Medical Products Agency published new recommendations for the cessation of prophylactic antibiotics in dentistry for the prevention of infective endocarditis (IE). Previously, 2 g of amoxicillin per os would be administered 1 h before invasive dental procedures to patients with valve prosthesis, complicated heart valve disease, and to those with previous endocarditis.

**Objectives:**

The aim of this study was to evaluate whether the total incidence of IE caused by oral viridans group streptococci (VGS) or IE caused by staphylococci, increased in Sweden after the introduction of the new recommendations.

**Methods:**

The incidence of IE in Sweden before and after October 2012 was calculated and compared using an interrupted time series analysis. Separate analyses were conducted for the total incidence of IE, and IE caused by VGS or *Staphylococcus aureus*. Cases of IE were identified using the Swedish national registry of IE, which has existed since 1995 and contains data from all Swedish hospital clinics specialising in infectious disease. All cases with hospital admission date from the 1^st^ of Jan 2008, to the 31^st^ of Dec 2017 were included. The incidence calculations were corrected for annual changes in population size using data from the Swedish government agency Statistics Sweden.

**Results:**

The results show no statistically significant increase in the slope of the trend line of the total incidence of IE, IE caused by VGS or *S. aureus* in the Swedish general population after October 2012, compared to before.

**Conclusion:**

The results suggest that the recommended cessation of prophylactic antibiotics for the prevention of IE in dentistry has not led to an increased incidence of IE caused by oral streptococci among the Swedish population.

## Introduction

Infective endocarditis (IE) arises when the innermost layer of the heart, the endocardium, is colonised by microorganisms circulating in the blood [1]. The colonisation is usually located to the heart valves, where microorganisms subsequently form a vegetation. As the disease progresses, it can lead to heart failure and embolization of blood vessels caused by circulating vegetation fragments [2]. The disease is fatal if left untreated and associated with an in-hospital mortality of 10–30% [1,3,4].

Treatment is focused on the eradication of microbes by antimicrobial treatment alone or in combination with surgery, and can require specialists in several medical fields such as infection, cardiology and thoracic surgery [1].

The most common pathogens causing IE are viridans group streptococci (VGS); a group of alpha-hemolytic bacteria commonly found in the oral cavity, and staphylococci (*Staphylococcus aureus* and *Staphylococcus epidermidis*), commonly found on the skin [5]. VGS cause a slower and less aggressive progression of IE, with lower mortality, than staphylococci [5,6].

The incidence of IE in Sweden has been reported to be 6.2–7.7 cases per 100 000 individuals per year [4,7]. Ternhag et al. found an increasing incidence of IE in Sweden from 1997 to 2007 [4]. Internationally, the incidence of IE ranges from 2 to 7 cases per 100 000 individuals per year [1,8].

Healthy endocardium is usually resistant to colonization, but disease and congenital malformations may result in damage of the endocardium causing a predisposition for IE [5]. Previous endocarditis, heart valve disease and prosthetic heart valves are important risk factors for IE. Elderly patients (>65 years of age) also have an elevated risk of IE. This may be due to the clustering of risk factors and the presence of undetected minor degenerative valve lesions [1]. However, 40% of the patients developing IE lack previously known heart disease [9–11].

The use of antibiotic prophylaxis (AP) in dentistry for the prevention of IE is controversial. The purpose is to reduce bacteraemia caused by invasive dental treatment and prevent colonization of the endocardium, thereby reducing the risk of IE [1]. However, the efficacy of the prophylaxis has not been confirmed [12]. Bacteraemia caused by VGS is known to occur not only after invasive dental treatment, but also during daily dental activities such as tooth brushing and flossing [13–17].

Current guidelines in Europe and America have restricted the indications for AP in dentistry, stating that it should be administered only to individuals with the highest risk of developing IE [2,18–20]. In Sweden, cessation of the prophylaxis to both low risk and high risk patients groups has been recommended since October 2012 [21].

In 2015, Thornhill et al. showed that a single dose of 3 grams of amoxicillin was associated with a low number of adverse drug reactions [22]. A restrictive approach towards the use of AP is however supported by data showing that one single dose of amoxicillin can induce significant disruption in the oral microbiota, and cause selection of bacteria resistant to antibiotics [23]. Antimicrobial resistance constitutes an enormous threat to modern healthcare, because numerous treatments require the use of effective antibiotics [24]. The association between the prescription of antibiotics and antimicrobial resistance is undisputed, and great effort is focused on reducing the use of antibiotics [25].

The benefits of a restrictive approach towards the use of antibiotics must be weighed against the potential risks. Considering the severity of IE it is of utmost importance to investigate the effect of refraining from AP.

The aim of this study was to evaluate whether the incidence of VGS-IE increased in Sweden after the introduction of new recommendations for AP in dentistry.

## Materials and methods

### Study design

An interrupted time series analysis (ITSA) was conducted of the monthly incidence of IE in the Swedish general population from 2008 to 2017, in order to calculate any increased incidence of the disease after the recommended cessation of AP in dentistry for the prevention of IE in October 2012. The change in recommendations allowed comparison of the incidence of IE caused by oral streptococci at a time when antibiotics was – and was not – recommended for the prevention of IE. Three separate analyses were conducted of the incidence of 1) all cases of IE, 2) IE caused by VGS, and 3) IE caused by *S. aureus*, in order to calculate and compare pre- and post-intervention trends. IE caused by VGS was considered to be of oral origin and thus of relevance with regard to cessation of AP during invasive dental procedures. The total incidence of IE and the incidence of IE caused by *S. aureus* were analysed to monitor trends in IE not related to the altered AP recommendations. Patients included in the study fulfilled the criteria for definite or possible IE according to the modified Duke criteria [26].

### Sources of data

Cases of IE were identified using the Swedish national registry of IE (SRIE), with an estimated coverage of 88% of Swedish cases of IE. Detailed information of the causative organism was available in 92% of cases. Age and sex distribution in the SRIE is displayed in Table 1, with the in-hospital mortality and proportion causative microorganisms.

**Table 1.** Characteristics in the Swedish national register for infective endocarditis (IE).

Period	Jan 2008 – Oct 2012		Oct 2012 – Dec 2017		Total	
	n	%	n	%	n	%
Age						
Mean	64.5		65.2		64.9	
Minimum	18		17		17	
Maximum	99		100		100	
Sex						
Female	677	34	824	31	1501	32
Male	1308	66	1840	69	3148	68
Total	1985	100	2664	100	4649	100
Causative microorganism						
VGS	497	25	653	25	1150	25
*S. aureus*	728	37	995	37	1723	37
Other	623	31	822	31	1445	31
Unknown	137	7	194	7	331	7
Total	1985	100	2664	100	4649	100
Mortality during treatment of IE						
VGS	28	6	38	6	66	6
*S. aureus*	115	16	113	13	248	14
Other	58	9	79	10	137	9
Unknown	8	6	15	8	23	7
Total	209	11	265	10	474	10

The table shows the in-hospital mortality of IE caused by viridans group streptococci (VGS), *Staphylococcus aureus*, and other microorganisms (mainly enterococci and coagulase-negative staphylococci). Data are displayed separately for the period before and after the cessation of antibiotics in dentistry for the prevention of IE, in October 2012.

In 1995, the Swedish Society for Infectious Diseases introduced a Swedish national registry of IE (the SRIE). All 30 departments of infectious diseases (ID) in Sweden have participated in the registry since its inception. These ID departments have regional responsibility for the care of patients with severe infections, and patients requiring acute surgery for IE are, in most cases, treated in ID departments during the pre- and/or postoperative period.

All cases are reported on a standardized questionnaire at the time of discharge and a second questionnaire after follow-up (mean: 3 months after treatment). Data regarding risk factors, presence of prosthetic valve, type of prosthetic valve and presence of other implantable cardiac devices is collected. The origin of the etiologic agent is verified using, for example, blood cultures, cultures from valves during surgery, and polymerase chain reaction (PCR) from tissue samples of valves. Information about antibiotic treatment, need for surgery, and treatment outcome is also included.

The registry has been used to present clinical data regarding various pathogens [27–32] and has made contributions to numerous studies within the framework of the International Collaboration on Endocarditis (ICE) with around 30 publications [3,6,33].

Children and young adults from the age of 0 to 16 years are treated at paediatric clinics not reporting to the register. However, the incidence of IE among children has been reported to be lower than among adults in Sweden [7,34]. We studied data from January 2008 to 2017 which covered an approximate period of 5-years before and 5-years after the recommendations to cease AP.

The incidence calculation was corrected for annual changes in the size of the Swedish population. The Swedish population, above 16 years of age, increased from 7 463 069 individuals in December (Dec) 2008, to 8 107 436 in Dec 2017. Data on population size was obtained from Statistics Sweden, the government agency responsible for developing, producing and disseminating official statistics and other government statistics in Sweden. Data are collected by Statistics Sweden on the 31^st^ of December each year, and is public and readily available in the statistical database on their website [35].

### Outcomes

The primary outcome measured in this study was the incidence of VGS-IE in Sweden. In addition to this, analyses were carried out on the total incidence of IE, as well as the incidence of IE caused by *S. aureus.*

### Statistical analysis

The statistical analysis was carried out using Stata/IC 15 (StataCorp. 2017. Stata Statistical Software: Release 15. College Station, TX: StataCorp LLC). We used the ITSA module (Ariel Linden, 2014. ‘ITSA: Stata module to perform interrupted time series analysis for single and multiple groups,’ Statistical Software Components S457793, Boston College Department of Economics, revised 8 December 2017) to perform a single group ITSA. The time of the intervention was set to month number 59, corresponding to October 2012, when the change in recommendations for AP in dentistry was published by the Swedish Medical Products Agency (MPA). Each point in the ITSA represents the monthly incidence of endocarditis.

### Ethics

This study was approved by the Regional Ethical Review Board, Karolinska Institutet, Stockholm, Sweden (ref: 2015/1910-31/1) prior to onset of the study.

An ethical permit and approval for the study was also obtained from the register board of the SRIE, before the collection of data.

Informed consent was waived because the study does not pose any risk of physical harm to subjects, no identifying information is published as results are presented on group level only.

## Results

Cases with known microbial aetiology not attributed to VGS or *S. aureus*, were mainly caused by enterococci (11%) and coagulase-negative staphylococci (6%).

**Figure 1.**
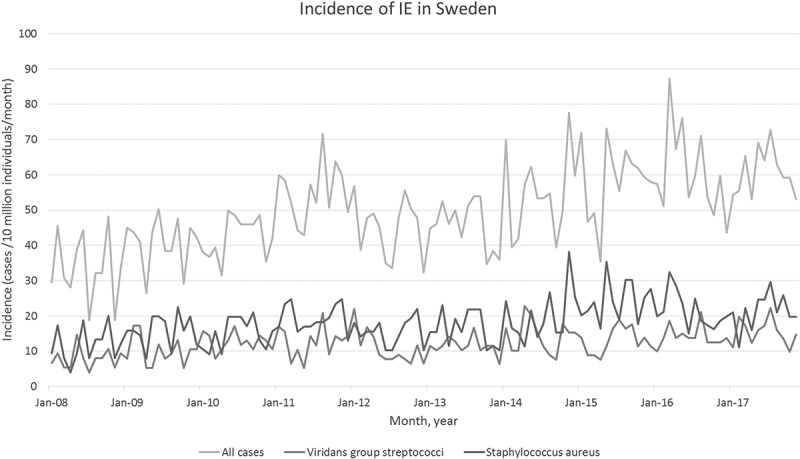
Incidence of infective endocarditis (IE) in Sweden.

Figure 1 shows the incidence of IE, by causative microorganism across the study period. From January (Jan) 2008 to Dec 2017, the incidence of VGS-IE in Sweden varies between 5.2 and 22.0 cases per 10 million individuals per month (Figure 1).

### IE caused by VGS

**Figure 2.**
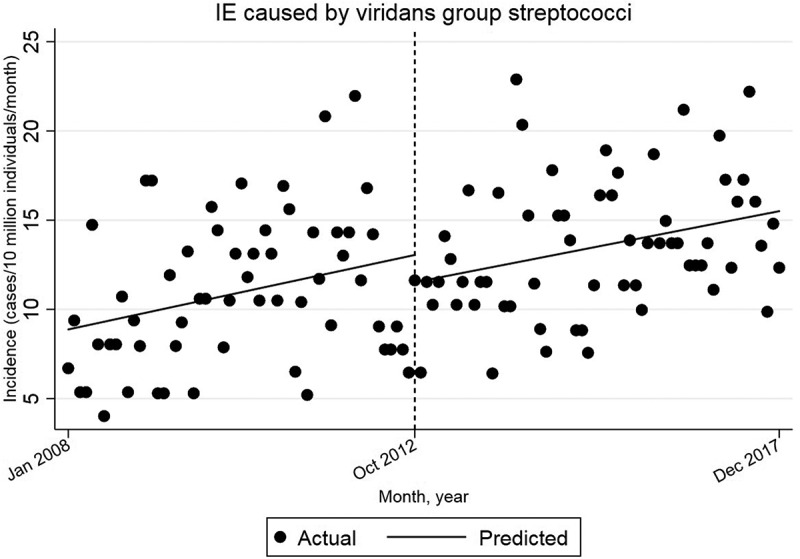
Single-group ITSA with Newey–West standard errors and one lag.

After October 2012 there is a statistically significant increase in the incidence of IE caused by VGS by 0.065 cases per 10 million individuals per month (95% CI = [0.017, 0.114]) (Figure 2).

Comparison with the pre-intervention trend yields a decrease in the slope of the trend line by −0.007 cases per 10 million individuals per month (95% CI = [−0.085, 0.082]) after October 2012. This decrease is not statistically significant.

The starting level is estimated to 8.9 cases/10 million individuals/month in Jan 2008. The incidence appears to increase by 0.072 cases per 10 million individuals per month (95% CI = [−0.002, 0.146]) until October 2012. This trend is not statistically significant.

Additional analyses were performed to account for lag in adherence to the new recommendations. Changing the time of intervention to 6, 12 or 18 months after October 2012 did not yield any statistically significant increase in the incidence of VGS-IE, compared to the pre-intervention trend.

### The total incidence of IE

**Figure 3.**
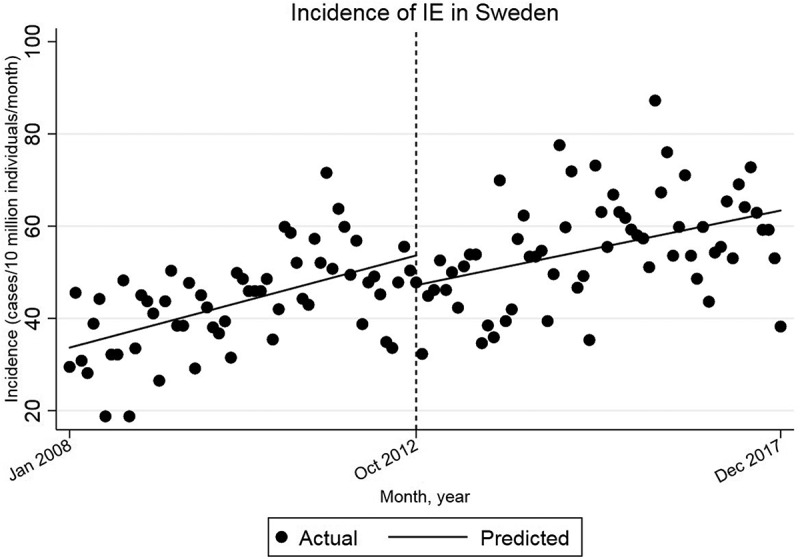
Single-group ITSA with Newey–West standard errors and one lag.

There is a statistically significant increase in the incidence of IE by 0.344 cases per 10 million individuals per month (95% CI = [0.187, 0.502]) before October 2012 (Figure 3). After October 2012, there is also a statistically significant increase in the incidence of IE, by 0.266 cases per 10 million individuals per month (95% CI = [0.115, 0.416]).

Comparison of the trends shows a decrease in the slope of the trend line after October 2012, relative to the pre-intervention trend, by −0.079 cases per 10 million individuals per month (95% CI = [−0.297, 0.139]). This decrease is not statistically significant. The starting level is estimated at 33.7 cases/10 million individuals/month in Jan 2008.

### *IE caused by* S. aureus

**Figure 4.**
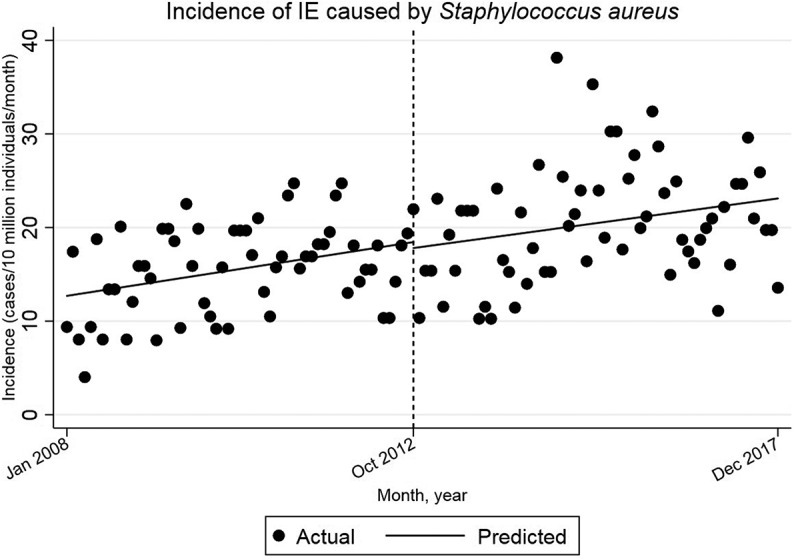
Single-group ITSA with Newey–West standard errors and one lag.

The incidence increases by 0.099 cases per 10 million individuals per month (95% CI = [0.022, 0.176]) until October 2012 (Figure 4). After October 2012, there is a decrease in the slope of the trend line, relative to the pre-intervention trend, by −0.012 cases per 10 million individuals per month (95% CI = [−0.121, 0.096]). This decrease is not statistically significant. The starting level is estimated at 12.7 cases/10 million individuals/month. Intervention is set to month 59, corresponding to October 2012. After October 2012 there is an increase in the incidence of IE caused by *S. aureus* by 0.087 cases per 10 million individuals per month (95% CI = [−0.011, 0.163]). This trend is not statistically significant.

## Discussion

To our knowledge, this is the first study of the incidence of VGS-IE following the recommended cessation of AP in Swedish dentistry for the prevention of IE in October 2012. The results imply that there was no increase, over the predicted positive trend, in the incidence of VGS-IE.

The UK and Sweden are the only countries to have abandoned AP to individuals considered to be at high risk of developing IE. Although in the UK, it is now emphasised that the patient should make an informed decision [36]. In Sweden, AP is in general prescribed by the dentist, and the dentist is ultimately responsible for the decision to administer or not to administer AP before dental procedures [21]. Previously in Sweden, 2 g of amoxicillin per os had been recommended 1 h before invasive dental procedures to patients with valve prosthesis, complicated heart valve disease, or previous endocarditis [21].

The number of prescriptions of amoxicillin by Swedish dentists decreased by 20% in 2013 compared to 2012 [37], and by 22.2% from 2013 to 2017 [38]. It has been suggested that the decrease may be due to the new recommendations [38].

The results of the current study show a statistically significant increasing trend in the total incidence of IE in Sweden both before and after October 2012. An increasing incidence of IE across the general population is in accordance with earlier studies of the epidemiology of IE in Sweden, USA, Italy, Taiwan and Canada [39, 40, 41, 4, Mackie et al.]. The increase may be due to increasing risk factors such as degenerative valve disease, receiving haemodialysis and population ageing [4,39–41].

The results are consistent with those reported by Thornhill et al in 2011 [42]. They excluded any statistically significant increase in the incidence of IE in England, during the 25 months after a cessation of AP.

In a later study however, Dayer et al. found an increased incidence over the projected trend [43] in England, and a 78.6% fall in AP prescribing. Although they did not have access to detailed data on the causative organism, and were unable to study the incidence of VGS-IE.

Current European and American guidelines have restricted the indications for AP in dentistry, stating that it should be administered only to individuals with the highest risk of developing IE [2,20]. Thornhill et al. published a review of studies monitoring the impact of these restricted guidelines [44]. They found that several studies were limited methodological flaws such as difficulties in accurately identifying IE caused by oral VGS and a lack of data on AP prescribing, and concluded that limiting AP use to those at highest risk seems pragmatic and appropriate.

Mackie et al. studied hospitalizations in Canada due to IE before and after the publication of the 2007 AHA recommendations [45]. They found an increasing trend before and after 2007. Comparison showed no significant increase after 2007. They used ICD codes to identify cases of streptococcal IE, but were unable to identify cases caused by oral streptococci.

The incidence of IE has been reported to be 2–7 cases per 100 000 individuals per year [1,8], which is in accordance with our results based on the SRIE; 4 to 7 cases per 100 000 individuals per year. Ternhag et al. utilized the Swedish patient register to calculate the incidence of IE in Sweden from 1997 to 2007. We found a similar male predominance among IE-patients as in their study (40.8% females and 59.2% males), but their incidence of IE was higher, at 7.7 per 100 000 individuals per year. Notably, our study was not based on the patient registry, but on the SRIE.

It is possible that a calculation using the patient registry could overestimate the incidence of cases of IE in Sweden [4], while our calculation could underestimate it. There are two reasons for this. The first is that the patient registry does not discriminate between definite, possible and rejected cases of IE according to the Duke criteria. For this reason, there is a risk of misclassification leading to the inclusion of rejected cases of IE. Secondly, the coverage of the patient registry is higher than that of the SRIE.

The National Board of Health and Welfare founded the patient registry in 1964, and it reached full national coverage in 1987 [46]. The coverage of the SRIE has been estimated to 88%.

One advantage of the SRIE is that it contains detailed information on the microbiological aetiology of cases of IE. For this reason, we were able to calculate the incidence of VGS-IE, which is not possible using the patient registry. Future research could integrate both registries to benefit from their respective advantages.

The short follow-up time after the change in recommendations is one limitation, as the timeframe may not be long enough to detect subtle effects on the frequency of cases of VGS-IE. Despite this, ongoing data monitoring is imperative after the change in recommendations in Swedish dentistry.

Another limitation is that the microbial aetiology is unknown in 7.7% of cases in the register. Reasons for this are mainly that no blood culture was performed, or that the performed blood culture showed negative results. Additional analysis in the SRIE showed that in 43% of cases with unknown aetiology, a blood culture was performed but the results were negative. Previous research concludes that in less than 50% of cases where the blood culture was negative, this could be attributed to the administration of antibiotics prior to the drawing of blood for a blood culture [7]. Some of these cases could have been caused by VGS, thereby resulting in an underestimation of the incidence of IE with possible oral origin. On the other hand the effect on our analysis should be negligible, as it is unlikely that the microbial aetiology for unknown cases should differ before and after 2012.

A concern is that the study included all patients regardless of high or low risk to develop IE after invasive dental treatment. A study of a high-risk population, such as valve prosthesis bearers, is desirable.

The proportion of IE 1995–2012 caused by VGS, recorded in SRIE, decreased from 35 to 25% and the proportion of IE caused by *S. aureus* increased from 30 to 40%. These trends are in analogy with trends in other countries with developed health care systems and can make the results difficult to interpret.

## Conclusions

The results suggest that the Swedish recommendations for the cessation of antibiotics in dentistry published in October 2012 have not lead to an increase in the incidence of IE in Sweden. Caution should be taken when interpreting the results, due to a short follow-up period, no specific analysis of high-risk groups, such as patients with prosthetic valves, and a limited number of cases.

## Data Availability

The study was conducted using national Swedish registries. The data that support the findings of this study are available from the SRIE, but restrictions apply to the availability of these data, which were used under license for the current study, and so are not publicly available. Data on population size used for the incidence calculations are readily available from Statistics Sweden.
